# Trajectory and progress of opioid agonist therapy programs in the Kyrgyz Republic

**DOI:** 10.36922/ghes.2536

**Published:** 2024-09-26

**Authors:** Amanda R. Liberman, Ainura Kurmanalieva, Damira I. Biibosunova, Natalya Shumskaya, Roman Ivasiy, Daniel J. Bromberg, Lynn M. Madden, Frederick L. Altice

**Affiliations:** 1 Section of Infectious Diseases, School of Medicine, Yale University, New Haven, Connecticut, United States of America; 2 AIDS Foundation East-West in the Kyrgyz Republic, Bishkek, Kyrgyzstan Republic; 3 Division of Global HIV and TB, CDC, Bishkek, Kyrgyzstan Republic; 4 Department of Epidemiology of Microbial Diseases, School of Public Health, Yale University, New Haven, Connecticut, United States of America; 5 Heidelberg Institute of Global Health, Faculty of Medicine and University Hospital, Heidelberg University, Heidelberg, Germany; 6 Department of Psychiatry and Psychotherapy (Campus Charité Mitte), Charité—Universitätsmedizin Berlin, Berlin, Germany; 7 APT Foundation, New Haven, Connecticut, United States of America

**Keywords:** Kyrgyz Republic, Methadone policy, Opioid use disorder, Opioid agonist therapy

## Abstract

The incidence and mortality of human immunodeficiency viruses (HIVs) are rising in Eastern Europe and Central Asia (EECA), particularly among people who inject drugs. Opioid agonist therapies (OATs), such as methadone or buprenorphine, are the most effective treatments for opioid use disorder and serve as a key HIV-prevention strategy in EECA. OAT uptake across the region, however, has been limited. The Kyrgyz Republic was the first Central Asian country to initiate OAT and remains a pioneer in the region. To understand the progression of OAT scale-up, all legislations related to drug policies or methadone in the Kyrgyz Republic were analyzed from the country’s founding to March 2023 and compared with policies in neighboring countries, particularly Kazakhstan and Tajikistan. Concurrently, local news coverage and policy reports were also assessed. OAT has been available in the Kyrgyz Republic since 2001, initially as a pilot project funded by international donors, and then with gradually increasing state support. Since its inception, the methadone program has evolved and influenced neighboring countries in Central Asia, despite numerous political challenges. The Kyrgyz Republic has expanded access to methadone, revised clinical protocols, and increased the number of distribution sites in communities and the carceral system to ensure broader access, aiming for program sustainability. While methadone policies and implementation in the Kyrgyz Republic have advanced earlier and more expansively than in neighboring countries, some challenges persist. Based on the findings, the suggestions provided may support the sustainable scale-up of methadone programs, enabling continued growth and improvement.

## 1. Background

While the incidence and mortality of human immunodeficiency viruses (HIV) have declined in other regions over the past decade, in Eastern Europe and Central Asia (EECA), HIV-related incidence and mortality are increasing (Nachega et al., 2023; UNAIDS, 2022a). The epidemic is driven by the injection of opioids, for which maintenance with opioid agonist therapies (OATs), such as methadone or buprenorphine, is among the most effective strategies (Alistar et al., 2011; Degenhardt et al., 2019a; Tan et al., 2019; Ward et al., 2022). A systematic review found that OAT is associated with an average 54% reduction in HIV incidence among people who inject drugs (PWID; MacArthur et al., 2012), and it is now considered the global standard of care for opioid use treatment (Degenhardt et al., 2019b). To effectively decrease HIV incidence among PWID, OAT should cover at least 20% and preferably 40% of PWID (World Health Organization [WHO] et al., 2009). In EECA, however, OAT coverage has remained minimal at best – many EECA countries do not offer this life-saving intervention and those that do often only provide it as pilot programs. In particular, the Russian Federation, a major influencer in the region, legislatively bans any form of OAT and seeks to influence its neighbors.

In the Kyrgyz Republic, the HIV prevalence among PWID is 60 times higher than that among the general population (18% vs. 0.3%; Joint UN Programme on HIV/AIDS, 2023). The Kyrgyz Republic is one of the few Central Asian countries to provide methadone, although coverage remains low at approximately 4.4% (UNAIDS, 2022b). This is comparable with other countries in EECA offering methadone: 7.1% in Ukraine, 0.4% in Kazakhstan, and 2.7% in Tajikistan. The Kyrgyz Republic was the first country to initiate OAT in Central Asia and has the most experience with this intervention in the region. Moreover, it is the first and only country in Central Asia to provide take-home dosing and OAT in the criminal justice system, with both programs still in operation. This article reviews the history and policies related to methadone in the Kyrgyz Republic, as well as potential barriers and suggestions for sustainable methadone scale-up in the country.

## 2. Methods

All laws relating to methadone, OAT, drugs/narcotics, and drug policy were reviewed from 1991 (the country’s founding) to March 2023 through the official legal databank of the Kyrgyz Republic (Ministry of Justice of the Kyrgyz Republic, n.d.). This analysis also included official reports from international funding organizations about the Kyrgyz methadone program. In addition, relevant laws governing methadone in the Kyrgyz Republic were compared with similar laws from the neighboring Kazakhstan and Tajikistan (Republic of Kazakhstan, n.d.; Republic of Tajikistan, n.d.). Notably, neither of the other two Central Asian countries (Uzbekistan and Turkmenistan) currently provide OAT, although methadone was briefly introduced and discontinued in Uzbekistan (Khachatrian, 2009). The Kyrgyz Republic, Kazakhstan, and Tajikistan all provide laws in Russian as well as in Kyrgyz, Kazakh, and Tajik, respectively; therefore, legal analyses were conducted in Russian by bilingual research team members (ARL, AK, RI, and DJB).

## 3. Results and discussion

**Figure 1.**
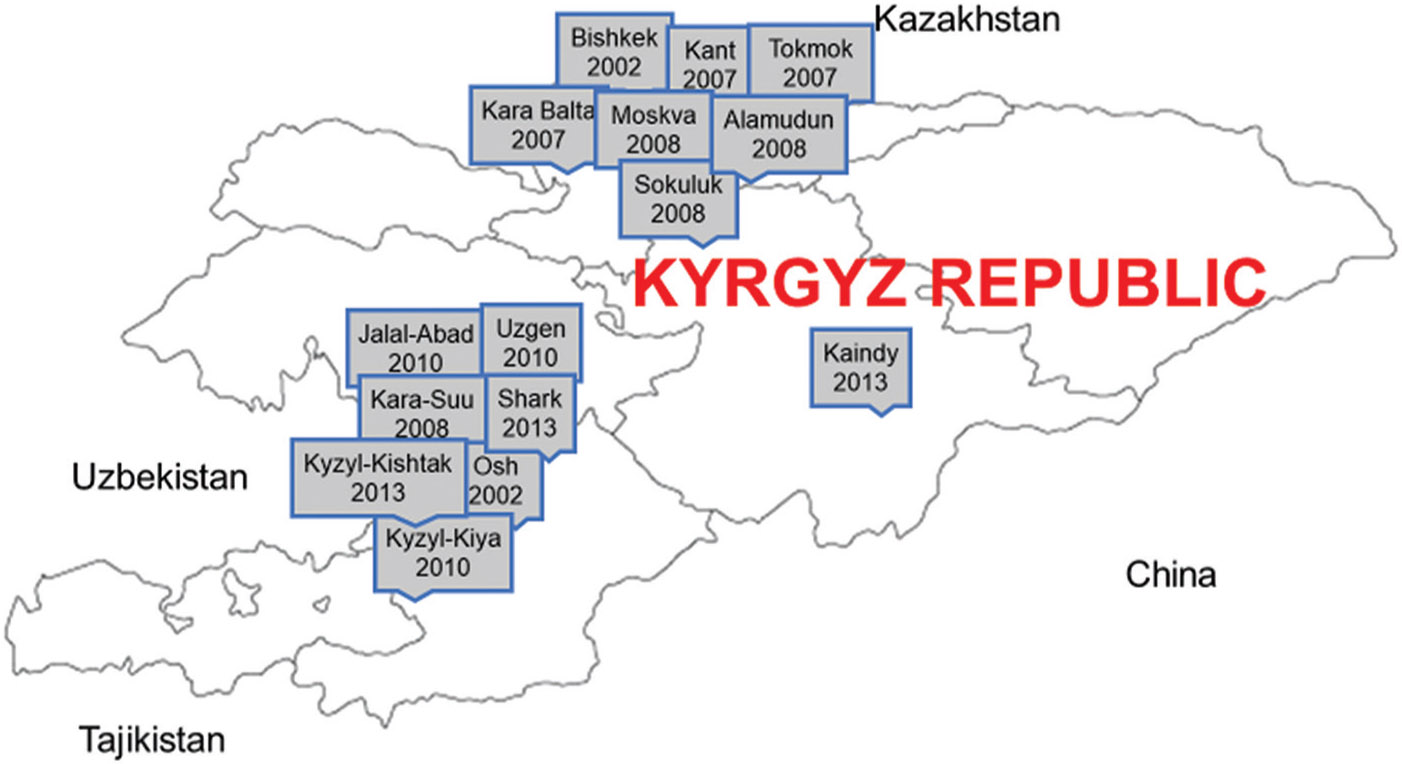
Map of non-penitentiary methadone sites in the Kyrgyz Republic and their start years. Image created by the authors.

**Figure 2.**
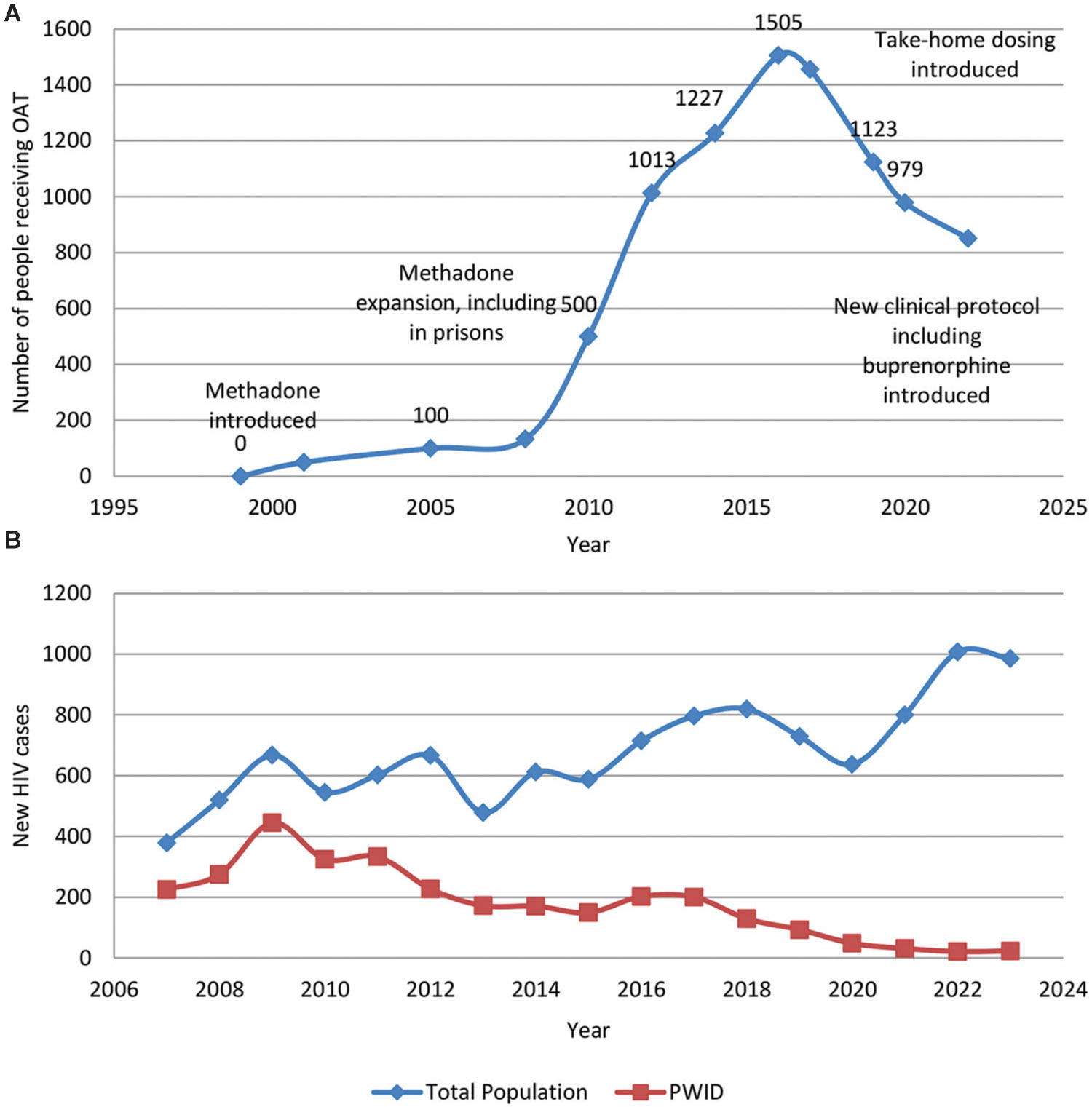
The number of new HIV cases among PWID decreases as OAT expands in the Kyrgyz Republic. (A) Total number of people receiving opioid agonist therapies by year in the Kyrgyz Republic. (B) Number of new HIV cases per year in the Kyrgyz Republic among the total population (shown in blue) and among people who inject drugs (PWID) (shown in orange) (Republican AIDS Center of the Ministry of Health of the Kyrgyz Republic, n.d.). In 2010, PWID accounted for nearly two-thirds of new HIV cases in the Kyrgyz Republic; by 2022, PWID represented only about 2% of new HIV cases.

Table 1 provides a history of key legislation and policy changes throughout the Kyrgyz Republic’s methadone program. Key moments include the approval and launch of the first OAT site in the Kyrgyz Republic in 2001, the development of the first formalized methadone clinical protocol in 2010, the expansion of take-home dosing to all methadone program participants in 2020, and the introduction of buprenorphine as an OAT modality in 2020. Figure 1 presents a map indicating when the first non-penitentiary methadone site opened in each Kyrgyz city. Figure 2A presents a timeline of the OAT census by year, and Figure 2B shows the number of new HIV cases per year in the Kyrgyz Republic among all individuals and PWID. From 2010 to 2022, PWID has gone from representing nearly two-thirds of new HIV cases to representing only about 2% of new cases (Republican AIDS Center of the Ministry of Health of the Kyrgyz Republic, n.d.). PWID began representing less than half of new HIV cases in 2012, at the same time as the rapid expansion of the OAT census in the Kyrgyz Republic.

### 3.1. Introduction of OAT into the criminal justice system

In August 2008, a pilot OAT program was launched in Kyrgyz Republic’s Colony No. 47, a penal institution, earning praise from the WHO for its organizational and clinical effectiveness, given the prevalence of within-prison drug injections (Azbel et al., 2018). This program led to reduced injection drug use among inmates, lowered risk of transmitting HIV, improved quality of life, and enhanced overall health (Subata et al., 2015). In 2009, two additional OAT sites were established, extending the program to the pretrial detention center in the Kyrgyz Republic. By June 2021, a total of 14 OAT sites were operational in prisons, serving nearly 400 patients (Republican Narcology Center, n.d.). The findings from the methadone program in Kyrgyz prisons showed early substantial scale-up and increased linkage to treatment after release, especially when patients were maintained on methadone at doses exceeding 80 mg/day (Bachireddy et al., 2022).

As described in Table 1, in 2015, clinical guidelines were released for in-prison or pretrial detention methadone treatment, covering post-admission and pre-release scenarios, such as treatment interruption. If the methadone treatment is interrupted by more than 2 days, patients must follow a treatment initiation protocol. Meanwhile, for interruptions of <2 days, patients can resume treatment at the previous dose after consulting with the site narcologist, a physician specializing in addiction treatment. One month before their release, patients must decide whether to continue treatment post-release. If they choose to discontinue the treatment, their clinician will follow a dose tapering protocol; meanwhile, those wishing to continue will be connected to a community methadone site (Republican Narcology Center, 2015).

### 3.2. Strategies to consider in other Central Asian contexts

The Kyrgyz Republic was an early adopter of methadone compared with other Central Asian countries, and it has the most experience in the region. In 2001, it became the first Central Asian country to offer methadone treatment, and in 2008, it became the first Central Asian country to offer methadone in prisons (on the extension of the State Program for Prevention of HIV/AIDS Epidemic and Its Socio-Economic Consequences in the Kyrgyz Republic for 2006-2010 until 2012, 2011); it is now approved but not implemented yet in Tajik prisons. OAT was expanded to pretrial detention (SIZO) in 2009 with the understanding that opioid use disorder (OUD) is a chronic, relapsing condition that should be continuously treated irrespective of location.

Ongoing collaborations between local and international organizations have resulted in multiple reports aimed at maximizing uptake and strengthening the country’s methadone program (Bachireddy et al., 2022; Ivasiy et al., 2022; Katkalova, 2021; Latypov et al., 2012; Liberman et al., 2021; Liberman et al., 2022; Moeller et al., 2009; Subata et al., 2015). The program’s strengths include sites in multiple regions within and outside of prisons, the integration of a one-stop-shop care model at certain sites (where individuals can receive HIV treatment and other health care at the site where they receive methadone), the recent addition of buprenorphine to clinical guidelines for OAT, and state and political support for the program. At present, funding for the program is divided between international organizations (Global Fund and PEPFAR) and the state budget, with plans to continue requesting international funding until at least 2026 (Kyrgyz Republic Funding Request Form, 2022). Notably, the Kyrgyz Republic is the only country in Central Asia to provide take-home dosing, which was expanded for methadone program participants in 2020 during the lockdowns related to the COVID-19 pandemic. It is the only Central Asian country to provide clinical guidelines for buprenorphine as OAT, which was introduced in 2022, thereby allowing patients to choose their preferred OAT.

The most recent clinical protocol for methadone dosing, developed in 2022, is an update from the previous protocol issued in 2015 (Ministry of Health of the Kyrgyz Republic and Republican Centre for Psychiatry and Narcology, 2022; Ministry of Health of the Kyrgyz Republic and Republican Narcology Center, 2015). The current guidelines reflect a review of OAT policies by domestic and international experts, as well as a review of the existing literature. The strengths of the updated protocol include the introduction of buprenorphine as an alternative to methadone, an emphasis on the importance of methadone for pregnant or breastfeeding individuals, appropriate dosing recommendations including early dose escalation, and no maximum treatment duration. The protocol provides updated guidance for transitioning patients between community and criminal justice settings, with the national OAT database allowing a patient to transition between sites. Although the protocol recommends psychological counseling for all methadone program participants, it does not require it, meaning that some patients may not receive psychological services alongside methadone treatment. Finally, the updated protocol recommends doses of at least 80 mg for most patients (previously 60 mg), though it allows for lower doses, and providers often prescribe subtherapeutic dosing (Ivasiy et al., 2022).

### 3.3. Ongoing challenges to OAT in the Kyrgyz Republic

Despite the strengths of the framework, the methadone program in the Kyrgyz Republic is currently declining in terms of the number of participants and program sites, as shown in Table 1 and Figure 2 (Katkalova, 2021). The decrease in the number of sites is mainly due to the consolidation of smaller sites into fewer, larger ones, whereas the decline in the number of participants is a more complex issue that is partially related to the introduction of stimulants, although opioids remain common (Katkalova, 2021). Notably, although the overall number of new cases of HIV cases in the Kyrgyz Republic has remained steady since the introduction of the OAT program, new HIV cases among PWID have dropped dramatically from 2010 to 2022 (Figure 2).

Table 2 summarizes the primary current legislative barriers to treatment scale-up and recommendations for sustainable program improvement. Subtherapeutic dosing of methadone, as described above, may also contribute to the declining number of participants. Despite efforts to reform law enforcement interactions with people enrolled in methadone programs (on Measures to Reform the System of Law Enforcement Agencies of the Kyrgyz Republic, 2016), police harassment of program participants remains common (Katkalova, 2021). Prescribed doses remain lower than those recommended by international guidelines (Ivasiy et al., 2022), and higher dosing is associated with better quality of care provision and increased willingness among patients to start and remain on methadone treatments (Farnum et al., 2021). Within prisons, individuals who wish to join the methadone program face significant social barriers (Liberman et al., 2021), including a social hierarchy that opposes methadone use and restricts access to work, food, and recreation incarcerated people (Azbel et al., 2022; Azbel and Altice, 2022; Meyer et al., 2020; Ponticiello et al., n.d.).

The Kyrgyz Republic’s methadone program in the Kyrgyz Republic has also encountered several political challenges. Most notably, a 2011 documentary titled “The Trap” demonized the program, leading to calls for its closure (Trilling, 2012). Despite this, the program continued to expand until 2017, when the number of participants began to decline.

Individuals seeking treatment for substance use disorders, including methadone for OUD, must first be diagnosed with OUD and then officially register with the government – —a process that can bar them from future employment opportunities or even from obtaining a driver’s license (Eurasian Harm Reduction Association, 2019; Regulations on the Rules and Procedure for Identifying, Registering and Recording in Public Health Institutions of the Kyrgyz Republic Persons Who Allow Non-Medical Consumption of Psychoactive Substances, 2001). Similar policies in other EECA countries have continued to hinder access to treatment (Bojko et al., 2013; 2015; Makarenko et al., 2016; Mazhnaya et al., 2016). By law, registered individuals are barred from working in state or municipal services, educational institutions, and taxi companies, among others (Eurasian Harm Reduction Association, 2019; Labor Codex of the Kyrgyz Republic, 2004). While the program aims to protect the community by preventing people who use psychoactive substances from operating heavy machinery or performing similar tasks, in practice, it creates a major barrier for individuals with substance use disorders to seek treatment. Before seeking treatment, a person may not be on the registry, but once a public clinic is aware of the person’s substance use disorder, registration is required, leading to the loss of their driver’s license and potential barriers to employment. In addition, once registered, it is difficult to be removed from the registry; although the laws and protocols dictating the registration process are detailed, the deregistration process is unclear.

The consequences of mandatory government registration have been discussed and analyzed extensively, with numerous publications identifying it as a barrier to treatment (Aizberg, 2008; Alieva et al., 2013; Eurasian Harm Reduction Association, 2019; Katkalova, 2021; Latypov et al., 2012; Subata et al., 2015; Yesenamanova et al., 2014). Despite these findings, the registration process remains in place.

### 3.4. Comparison to neighboring countries

The Kyrgyz Republic has a strong, state-supported OAT program, although with several challenges, as outlined above. Its enabling framework is considerably stronger than programs in neighboring countries. Russia, Turkmenistan, and Uzbekistan do not currently have methadone programs–methadone and all other OAT programs are explicitly banned. Uzbekistan introduced methadone but discontinued the pilot program in 2009 (Khachatrian, 2009). Meanwhile, programs in Kazakhstan and Tajikistan are exceptionally small in terms of coverage, even when compared to the Kyrgyz Republic’s suboptimal coverage rate of 4.4% (UNAIDS, 2022b). Similar to the Kyrgyz Republic, Kazakhstan and Tajikistan require mandatory registration for PWID. The methadone programs in Kazakhstan and Tajikistan also face numerous infrastructure challenges. Kazakhstan, for example, has struggled with procurement issues, leading to several program shutdowns (Kazakhstan Union for PLHIV et al., n.d.), endangering the lives of patients. In Tajikistan, the methadone program is limited to a few sites in major urban centers, restricting access for much of the population (Kluczewska and Korneev, 2021).

## 4. Conclusion

This article provides a review and timeline of the major legislative benchmarks in the Kyrgyz Republic’s OAT program, highlighting the stepwise policy changes that have supported the program’s sustainability since its inception as a pilot in 2001. It also identifies areas where improvements can be made to ensure the program’s sustainability. The analysis included all laws relating to methadone, OATs, drugs/narcotics, and drug policies in the Kyrgyz Republic from its founding in 1991 to March 2023, as well as comparisons with OAT programs in other countries in the region. The article traces the trajectories of OAT scale-up in relation to overall HIV transmission, in particular, among PWID. It is evident that the Kyrgyz Republic’s legislative framework has been more supportive than those of other countries in the region. Moving forward, implementing changes such as ensuring optimal methadone dosing (at least 80 mg per day), expanding access to buprenorphine, eliminating mandatory narcological registration, and improving law enforcement interactions with OAT participants can help sustain the Kyrgyz OAT program and provide a model for other countries in Central Asia.

## Figures and Tables

**Table 1.** Timeline for key legislative and policy related to methadone

Year	Policy change	Activities	OAT Census (Sites)	Source
1998	Order 66	Establishes regulation of narcotics. Methadone remains on the list of controlled narcotic substances as of 2019. Clarifies that addiction is a disease, framing people who use opioids as patients instead of criminals.	0 (0)	(About Narcotic Drugs, Psychotropic Substances and Precursors, 1998; On Amendments to the Resolution Government of the Kyrgyz Republic “On Narcotic Drugs, Psychotropic Substances, and Precursors Subject to Control in the Kyrgyz Republic” Dated 9 November 2007 No. 543, 2018)
1999	Order 60	Establishes citizens’ right to quality mental health care, including treatment for substance use.	0 (0)	(On Psychiatric Care and Guarantees of Citizens’ Rights in the Provision of Psychiatric Care, 1999)
2001	Orders 41, 71	Approves and launches the first OAT site in Bishkek for HIV prevention. Sets conditions for program entry, such as two prior hospitalizations for OUD, no polysubstance use, and the prospective participant’s agreement not to leave the city for at least a year.	50 (1)	(On Amendments to Order No. 71 of 2001 March 13 on the Conditions and Procedure for Substitution Therapy for Persons with Drug Dependence in the Kyrgyz Republic, 2002; Program “Methadone Substitution Therapy in Bishkek”, 2001)
2002	Order 17	Opened the second OAT site in Osh and includes provisions for future program expansion in terms of numbers and sites.	50 (2)	(On Amendments to Order No. 71 of 2001 March 13 on the Conditions and Procedure for Substitution Therapy for Persons with Drug Dependence in the Kyrgyz Republic, 2002)
2005	Orders 6, 82, 149	Establishes health-care protections for PWH. Laws allow law enforcement to mandate the registration of dependent persons, linking substance use treatment for PWH and PWID.	100 (2)	(On HIV/AIDS in the Kyrgyz Republic, 2005; On the Prevention of Offences in the Kyrgyz Republic, 2005; On the Protection of Citizens’ Health in the Kyrgyz Republic, 2005; Trends and Events in Harm Reduction in 2005: Countries with Predominantly Injection-Driven HIV Epidemics, 2006)
2006	Order 498, 759	Establishes a State Program for the Prevention of the HIV/AIDS Epidemic, supporting OAT expansion and allowing OAT introduction in prisons. Methadone is added to the essential medicines list in the Kyrgyz Republic, despite being on a list of dangerous drugs subject to national control.	100 (2)	(On the Extension of the State Program for Prevention of HIV/AIDS Epidemic and Its Socio-Economic Consequences in the Kyrgyz Republic for 2006-2010 until 2012, 2011)
2007-2008	Orders 15, 56	Further expands the methadone program by creating additional sites, including the first prison-based sites.	133 (16)	(On Expanding the Program of Substitution Maintenance Therapy with Methadone for Opioid Dependence in the Territory of Bishkek and Chui Region, 2011; On the Expansion of the Methadone Substitution Treatment Program in the Branches of the Organizations of the Ministry of Health of the Kyrgyz Republic, 2007)
2010	Order 494, Order 497	Permits a naloxone program for overdose prevention. The first formal methadone clinical protocol is developed, requiring daily clinic attendance with strict guidelines.	500 (20)	(Ministry of Health of the Kyrgyz Republic and Republican Narcology Center, 2015; On the Implementation of the Project GLO J71/OFID-UNODC, 2010)
2012	Order 703	Develop official clinical guidelines for OUD treatment, including methadone.	1013 (20)	(Ministry of Health of the Kyrgyz Republic and Republican Narcology Center, 2017)
2014	Orders 91-r and 54	Order 91-r prohibits arbitrary detentions and searches of people who use drugs. Order 54 introduces an anti-drug program that includes OAT as a modality for secondary HIV prevention.	1227 (20)	(Matieva et al., 2015; On Approval of the Anti-Drug Program, 2014; On Approval of the Fifth National Report of the Kyrgyz Republic on the Implementation of the International Covenant on Economic, Social and Cultural Rights from 2012 to 2019 Years, 2021)
2015	Order 372	Creates a working group to revise the methadone treatment clinical protocol. New guidelines recommend maintenance doses as low as 60 mg for most patients and prohibit take-home dosing.	Approx. 1200 (31)	(Instructions on Implementation of Methadone Maintenance Therapy and Needle and Syringe Programs in Institutions of the State Penitentiary Service under the Government of the Kyrgyz Republic, 2015; On the Creation of a Working Group for the Revision of the Clinical Protocol “Treatment of Opioid Dependence Based on Maintenance Therapy with Methadone”, 2015; Republican Narcology Center, 2015; Subata et al., 2015)
2016	Orders 161, 637	Order 637 abolishes the State Drug Control Service, a law enforcement branch focused on trafficking and drug use through punitive measures. Order 161 establishes that illicit drug trafficking will be regulated by the Ministry of Internal Affairs and legal circulation of narcotic drugs including methadone will be regulated by the Ministry of Health. This creates a more public-health-based approach to drug regulation. In 2016, the government produced its first training manual for dispensing methadone by nurses.	1505 (31)	(On Additional Measures for Counteraction of Illicit Trafficking of Narcotic Drugs, Psychotropic Substances and Precursors, 2011; On Measures to Reform the System of Law Enforcement Agencies of the Kyrgyz Republic, 2016; On the Liquidation of the State Drug Control Service under the Government of the Kyrgyz Republic, 2016; Republican Narcology Center of the Ministry of Health of the Kyrgyz Republic, 2016)
2017	Orders 584, 625, 1082, 131	Order 584 introduces a clinical protocol for children and adolescents with substance use disorders, allowing treatment of OUD in minors. Order 625 allows oversight of the methadone program. Order 1082 allows methadone treatment in inpatient facilities and introduces take-home dosing for select patients for up to five days, and Order 131 provides guidance on how to implement Orders 625 and 1082.	1455 (29)	(On Amendments to the Regulations “On the Conditions and Procedure for Conducting Methadone Maintenance Therapy for Persons Who Inject Drugs in the Kyrgyz Republic”, 2017; On the Conditions and Procedure for Conducting Methadone Maintenance Therapy for People Who Use Injection Drugs in the Kyrgyz Republic, 2017; On the Implementation of Orders of the Ministry of Health of the Kyrgyz Republic No. 625 Dated July 17, 2017, and No. 1082 Dated November 28, 2017, 2017; Republican Narcology Center of the Ministry of Health of the Kyrgyz Republic, 2017)
2019	Orders 542, 749	Permit PWH to seek HIV services outside of specialized clinics, including receiving antiretroviral medications at methadone sites. These orders also provide a clinical protocol for people who use opioids and alcohol.	1123 (24)	(On Approval of Mechanisms for Decentralization of Medical Services for People Living with Human Immunodeficiency Virus in the Kyrgyz Republic, 2019; Republican Narcology Center of the Ministry of Health of the Kyrgyz Republic, 2019)
2020	Order 28	Expands take-home dosing of methadone during the COVID-19 pandemic to mitigate community transmission of COVID. Allows take-home dosing for up to five days for patients whose relatives apply on their behalf with identifying documents.	979 (24)	(On Measures to Prevent Coronavirus Infections (COVID-19), 2020)
2022	Order 1471	Updates official guidelines for OAT, incorporating input from international experts. The updated guidelines target higher dosing, earlier transition to take-home dosing, and faster induction strategies. For the first time, guidelines for buprenorphine treatment are introduced.	850 (24)	(Ministry of Health of the Kyrgyz Republic and Republican Centre for Psychiatry and Narcology, 2022)

Abbreviations: HIV: Human immunodeficiency virus; OAT: Opioid agonist therapies; OUD: Opioid use disorder; PWH: People with HIV; PWID: People who inject drugs.

**Table 2.** Recommendations for reducing barriers to opioid agonist therapies and supporting sustainable scale-up of treatment

Observed barrier	Recommendation
Requirement for daily in-person treatment	1. Add sublingual and/or long-acting injectable buprenorphine to the national formulary. 2. Expand take-home dosing options, as during the COVID-19 pandemic, and allow longer periods of take-home self-administration.
Subtherapeutic methadone doses among many program participants (Ivasiy et al., 2022)	Encourage providers to prescribe higher doses, as indicated in the new 2022 clinical protocol, particularly for patients receiving treatment for HIV or tuberculosis.
Dilution of liquid methadone causes logistical shipping difficulties	1. Add sublingual buprenorphine to the national formulary. 2. Allow transport of more concentrated methadone formulations. 3. Allow tablets rather than liquid formulations.
Lack of OAT site accessibility and possible migration to areas without methadone sites	1. Allow any licensed prescriber to prescribe OAT, perhaps with limited training. 2. Expand the types of clinics allowed to dispense OAT (e.g., primary care clinics). 3. Create clear guidelines for transitioning into and out of the program to account for internal and external migration (e.g., if people go to work in the Russian Federation or another place without methadone). 4. Allow longer take-home dosing options for people who live remotely from OAT sites.
Patient fears of consequences of registration	Remove the registry entirely or establish clear guidelines for removing stable methadone patients from the narcological registry.
Police harassment of program participants	1. Incentivize police to refer people who inject drugs to needle/syringe exchange programs and/or OAT. 2. Foster partnerships between methadone programs and local police departments. 3. Train police on harm reduction and the benefits of OAT for public safety.

Abbreviation: OAT: Opioid agonist therapies.
